# Anatomical adaptations and ionic homeostasis in aquatic halophyte *Cyperus laevigatus* L. Under high salinities

**DOI:** 10.1016/j.sjbs.2021.03.002

**Published:** 2021-03-13

**Authors:** Sahar Mumtaz, Muhammad Hamzah Saleem, Mansoor Hameed, Fatima Batool, Abida Parveen, Syeda Fasiha Amjad, Athar Mahmood, Muhammad Arfan, Shakeel Ahmed, Humaira Yasmin, Abdulaziz Abdullah Alsahli, Mohammed Nasser Alyemeni

**Affiliations:** a Department of Botany, Division of Science and Technology, University of Education, Lahore 54770, Pakistan; b College of Plant Science and Technology, Huazhong Agricultural University, Wuhan 430070, China; c Department of Botany, University of Agriculture, Faisalabad 38000, Pakistan; d Department of Botany, Government College University, Faisalabad 38000, Pakistan; e Department of Agronomy, University of Agriculture, Faisalabad 38000, Pakistan; f Instituto de Farmacia, Facultad de Ciencias, Universidad Austral de Chile, Valdivia 5110566, Chile; g Department of Bio-Sciences, COMSATS University, Islamabad 45550, Pakistan; h Department of Botany and Microbiology, College of Science, King Saud University, Riyadh 11451, Saudi Arabia

**Keywords:** Aerenchyma, Plasticity, Sclerification, Osmoprotectants, Salt marshes

## Abstract

Salinity is extremely hazardous to agriculture worldwide and its expanding constantly. Soil of almost 100 countries facing salinity problem including Pakistan. *Cyperus laevigatus* also act as salinity indicator species is a naturally adapted halophyte dispersed in subtropical regions of world. Six populations of *C. laevigatus* were collected from different saline habitats to evaluate adaptations regarding anatomical and physiological characteristics. *C. laevigatus* is perfectly adapted to harsh environmental conditions like dry barren soils, saline lakes, hyper-saline wetlands and salt marshes. Ecological success of this species is due to plasticity in physiological and anatomical characteristics to adapt variable environmental conditions. *C. laevigatus* is a halophyte, exhibited increased biomass production in moderately saline habitat. Higher uptake of K^+^ occurs to compensate the uptake of Na^+^ ion contents, a striking feature of salt-tolerant and halophytic species. Accumulation of osmoprotectants like proline, free amino acids, soluble sugar and protein contribute significantly to osmotic adjustment. Stem thickness enhanced as salinity level of habitat increased to store water in parenchymatous tissues under physiological drought. Intensive sclerification in root cortex provide mechanical strength to plant as well as prevent the radial leakage of water. Well-developed aerenchyma, increased vascular bundle area, broader vessels, small and dense stomata are critical to cope with environmental hazards. Population of Jahlar lake showing maximum biomass production indicate that this species grows better in moderate salinities. Therefore, this species will prove very useful for revegetation of salt affected rangeland and prairies by direct growth of such halophytic ecotypes.

## 1 Introduction

Salinity is one of the major challenges for agricultural crops due to its effect on yield and sustainability particularly in arid and semi-arid regions of world (Iqbal et al., 2015, Ahmad et al., 2019, Adhikari et al., 2020). During 19th century, salinity emerged as leading problem in Pakistan due to limited crop production and loss in economy (Safdar et al., 2019). Salt-affected habitats like saline drylands and salt wetlands in Pakistan recognized by the presence of salinity indicator species such as *Cyperus lavigatus* (Khan and Qaiser, 2006)*.* In other regions of the world common salt indicator species are *Sporobolus virginicus, Juncus acutus, Salsola vermiculata, Salicornia europaea* and *Suaeda australis* (Aslamsup et al., 2011). Saline soil imparts several harmful effects on plants growth and development because of reduced water uptake and excessive ions absorbance, which ultimately leads to side effects on cellular level (Alam et al., 2020). Salinity can cause disturbance in nutritional requirement of plants (Saleem et al., 2019, Ahanger et al., 2020, Ali et al., 2020a, Kaya et al., 2020). NaCl stress is one of the most abundant because saline soils are dominated with high contents of Na^+^ and Cl^-^ those are found in excessive amount than plants requirement (Alam et al., 2019, Safdar et al., 2019). The higher quantity of salt in rhizosphere leads to the disturbance in aqueous and ionic balance like toxicity, ionic disparity and hyper-osmotic stress (Parihar et al., 2015). Due to these disturbances, enzyme activities and plant chemical reactions affect adversely (Liu et al., 2020, Rehman et al., 2020a, Saleem et al., 2020b, Saleem et al., 2020d, Saleem et al., 2020e).

Halophytes can endure higher concentrations of salt and have ability to flourish in stressed environment by accumulation of some important ions and osmolytes (Usman et al., 2018). Membrane integrity, K^+^/Na^+^ selectivity and osmoregulation maintained by higher contents of Ca^2+^ in plants growing under salt stress (Safdar et al., 2019, Yaseen et al., 2020). Soluble proteins, sugars and other solutes are crucial in osmoregulation such as enhanced water uptake and retention, protection of macromolecules structures which can be damaged under salt stress (Jabeen and Ahmad, 2017, Saleem et al., 2020c). Salt stress can cause adverse effects on plant morphological and anatomical features such as stunted root and shoot growth, reduced fruit and vegetable yield, shrinkage in stem diameter, smaller root cortex and inhibition of vascular growth (Iqbal et al., 2015, Zafar et al., 2015). Halophytes tolerate salinity stress by the development of specific anatomical structures like succulence in shoot and midrib, development of aerenchyma, larger vascular bundles, increased phloem and metaxylem area and intensive sclerification (Parida and Das, 2005, Imran et al., 2019, Mohamed et al., 2020a). Stomatal area, density and orientation contribute a lot in process of salinity tolerance (Mohamed et al., 2020b).

*Cyperus laevigatus* L. also known as smooth flat sedge is a perennial sedge dispersed in subtropical regions of world with hot climate. It flourishes mostly in aquatic habitats such as waterlogged soil, brackish water, coastal areas, flood plains and mud flats (Badr et al., 2020). This species used for the treatment of wetland ecosystems where NH_4_^+^ concentrations are high (Gamal et al., 2015). The objective of the present study was to evaluate adaptive components of salinity tolerance in differently adapted populations of *Cyperus laevigatus.*

## 2 Materials and methods

### 2.1 Collection of samples

**Fig. 1:**
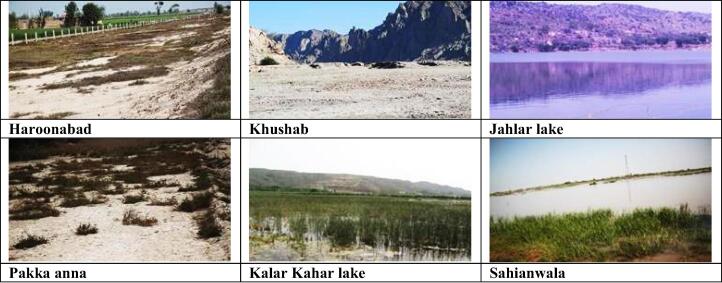
Pictorial description of saline collection sites of *Cyperus laevigatus.*

Six populations of *Cyperus laevigatus* were collected from different ecological regions of the province Punjab, viz., Haroonabad, Khushab, Jahlar Lake, Pakka anna, Kalar Kahar Lake and Sahianwala. These regions were selected on the basis of widespresd distribution of this species. The physico-chemical properties from rhizosphere of Cyperaceae species collection sites in the Punjab are presented in Table 1 & Fig. 1.

**Table 1:** Soil physicochemical characteristics from rhizosphere of Cyperaceae species collection sites in the Punjab.

Habitat	Har	Khu	Jha	PA	KK	Sah
**pH**	9.2	7.3	7.4	8.5	7.8	8.3
**ECe (dS m^−1^)**	3.4	14.2	16.8	32.7	37.3	46.3
**SP**	33.7	38.4	35.2	32.3	36.8	32.3
**Na^+^ (mg g^−1^ d.wt.)**	177.4	3210.5	2844.6	3733.7	4023.7	5759.1
**K^+^ (mg g^−1^ d.wt.)**	58.7	255.8	249.3	345.2	173.6	371.6
**Ca^2+^ (mg g^−1^ d.wt.)**	108.7	155.3	277.6	68.9	133.7	366.9
**Cl^-^ (mg g^−1^ d.wt.)**	433.2	1390.1	1611.8	1922.4	2029.8	2528.1
**Annual rainfall (mm)**	191	395	450	310	485	375
**Elevation (m a.s.l.)**	178	174	950	172.6	734.2	190.4
**Coordinates**	29° 60′ 81″ N 73° 14′ 68″ E	32° 17′ 55″ N 72° 21′ 3″ E	33° 38′ 17″ N 72° 20′ 38″ E	31° 15′ 63″ N 71° 50′ 19″ E	32° 46′14″ N 72° 42′ 32″ E	31° 39′ 47″ N 73° 13′ 31″ E
**Habitat description**	Situated on edge of Cholistan desert, sandy soil with drought tolerant vegetation, hot climate	Dry barren hills with sandy soil, very sparse vegetation mostly salt-tolerant	Salt-water lake situated in mountains, dominated by sedges and salt-tolerant grasses, cooler climate	Hyper-saline wetland, reclaimed by *Leptochloa fusca.*	Hyper-saline lake surrounded by hills, dominated by halophytes, cooler climate	Situated near Faisalabad, highly saline waterlogged area dominated with salt-tolerant and halophytic species

### 2.2 Shoot water relations

Scholar-type pressure chamber was utilized for the determination of shoot water potential of each replicate of six habitats. For the determination of osmotic potential same shoot was frozen at −20 °C for one week. Then frozen shoot was thawed, and cell sap extracted to determine osmotic potential by using vapor pressure osmometer (Wescor 5500). Value of turgor potential obtained by subtracting osmotic potential from the water potential.

### 2.3 Plant ionic content

0.1 g of shoot and root material from each sample was dried and ground, then it was subjected to digestion with conc. H_2_SO_4_ following Wolf (Wolf, 1982) to record the ionic contents values. Na^+^, K^+^ and Ca^2+^ were determined by flame photometer (Jenway, PFP-7).

### 2.4 Organic osmolytes

Total amino acid was determined according to the method used by Moor and Stein (Moore and Stein, 1948). 1.0 g fresh leaves chopped into 10 mL citrate buffer, incubated at room temperature for one hour and then centrifuged at 15000 rpm for 10 min at 15 °C. The supernatant then separated and used for the determination of free amino acids. 1.0 mL extract and ninhydrin solutions were added in vessel, covered with aluminum foil, and heated for 20 min in boiling water bath. The vessel was then cooled, 5 mL diluent added and subjected to incubation for 15 min at room temperature. OD was recorded at 570 nm on a UV–visible spectrophotometer (Hitachi 220, Japan).

To determine the total soluble proteins method of Lowry et al. (Lowry et al., 1951) was used. 0.2 g fresh leaves chopped in 5 mL 0.2 M phosphate buffer (7.0 pH). Chopped leaf material was then centrifuged at 5000 rpm for 5 min. 1.0 mL of supernatant from each sample and copper reagents added in a vessel, mixed thoroughly, and kept still for 10 min at room temperature. After that 0.5 mL of Folin-phenol reagent taken, mixed and then subjected to incubation for 30 min at room temperature. OD was recorded at 620 nm on a spectrophotometer (Hitachi 220, Japan).

Yemm and willis (Yemm and Willis, 1954) method was used for the determination of total soluble sugars. 0.1 g of fresh plant undergone extraction with 80% ethanol. The extracted material was kept in incubator at 60 °C for 6 h. This material was used to estimate the quantity of soluble sugars. The extract was mixed with anthrone reagent (6 mL) and then warmed for 10 min in boiling water. This was subjected to cooling with ice cubes for 10 min and then kept in incubator for 20 min at 25 °C. Optical density was observed at the wavelength of 625 nm on a spectrophotometer (Hitatchi, 220, Japan).

The estimation of proline contents was analyzed according to method performed by Bates et al. (Bates et al., 1973). 0.5-gram leaves mixed and homogenized with 3% sulfo-salicylic acid (10 mL). The resulting mixture filtered by Whatman no. 2 filter paper. 2 mL of acid ninhydrin solution (1.25 g ninhydrin in 30 mL glacial acetic acid), 2 mL of glacial acetic acid and 20 mL of 6 M orthophosphoric acid reacted with 2 mL filtrate at 100 °C for one hour. Ice bath used to complete this reaction. Product of this reaction was mixed with toluene (4 mL), and then fiercely mixed while air is passing through it for 1–2 min. After that toluene was extracted from this reaction, heated at 25 °C and absorbance was observed at 520 nm.

### 2.5 Anatomical parameters

**Fig. 2:**
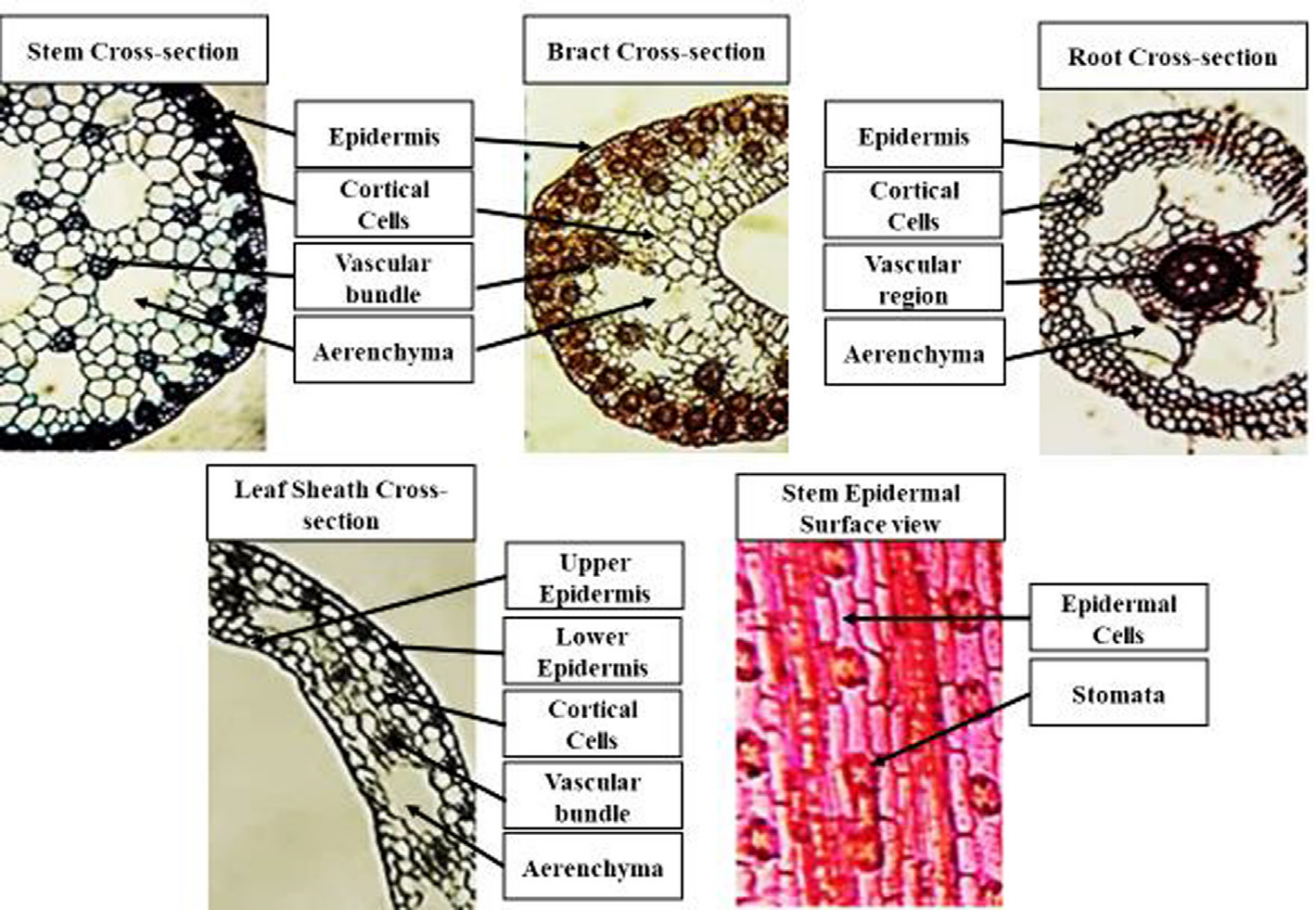
**Measurement detail of root, stem and bract anatomical characteristics of *Cyperus laevigatus*. a. Haroonabad Root TS:** Vascular region much reduced, cortical region comprising large regularly arranged aerenchyma. Outer cortex 3–4 layered thick. **b. Haroonabad Stem TS:** Average sized vascular bundles, large aerenchyma, densely packed chlorenchyma inside epidermis. **c.** Smaller vascular region with 6 narrow vessels, larger aerenchyma, outer cotex 4–6 layered thick, inner cortex much reduced. **d. Khushab Stem TS:** Small vascular bundles, average sized aerenchyma, higher sclerification inside epidermis. **e. Jhalar lake Root TS:** Vascular region with four broader vessels, average sized aerenchyma, cells of outer and inner cortex regularly arranged. **f. Jhalar lake Stem TS:** Smaller vascular bundles, regularly arranged aerenchyma and cortical cells, reduced chlorenchyma. **g. Pakka anna Root TS:** Vascular region with 3 broader and one narrow vessel, rare aerenchyma, smaller cortical cells. **h. Pakka anna Stem TS:** Larger vascular bundles, reduced aerenchyma, regularly arranged cortical cells, dense chlorenchyma. **i. Kalar Kahar lake Root TS:** Vascular region with 4 broadest vessels, very large aerenchyma, small sized cortical cells. **j. Kalar Kahar lake Stem TS:** Larger vascular bundles, irregular aerenchyma, chlorenchyma arranged inner side of epidermis sparsely. **k. Sahianwala Root TS:** Vascular region with 3 broader and one narrow vessel, smaller sized cortical cells near vascular region that become larger in mid and small cells inside epidermis. **l. Sahianwala Stem TS:** Medium to large vascular bundles, very large aerenchyma.

For anatomical investigation root, stem, bract and leaves were washed and preserved in (FAA) formalin acetic alcohol. Free hand sectioning technique was used to prepare permanent slides. Dehydration of transverse and epidermal sections was processed through series of ethanol grades. Standard double-staining procedure was adopted for staining the sections using safranin and fast green stains to differentiate between lignified and other tissues. Photographs of these slides was taken by camera-equipped compound microscope (Nikon 104, Japan). Anatomical data were recorded by ocular micrometer (Fig. 2).

### 2.6 Statistical analysis

Samples for anatomical studies were collected from three different sites of each habitat and then data were subjected to analysis of variance (one-way ANOVA) using Microsoft Exceland RDA (Redundancy analysis) using XLSTAT. The heat-map analysis between various variables are constructed using RStudio.

## 3 Results

### 3.1 Morphological characteristics

Shoot fresh weight showed a notable variation in populations of *C. laevigatus* collected from different habitats of Punjab. The highest shoot biomass was observed in those populations which were growing in Jhalar Lake. The lowest biomass was recorded in population of Pakka Anna. There was a significantly varied response of root fresh weight among habitats as the maximum weight was reported in Jhalar Lake, while population of salt marsh like Pakka Anna showed the minimum growth (Table 2).

**Table 2:** Morpho-physiological characteristics of *Cyperus laevigatus* collected from different ecological regions of Punjab.

	Har	Khu	Jha	PA	KK	Sah	F-ratio
**Morphological characteristics**							
Shoot fresh weight (g plant^−1^)	10.8b	9.2c	14.9a	8.7d	10.7bc	10.4bc	366.2***
Root fresh weight (g plant^−1^)	0.96d	1.2bc	4.1a	1.3b	1.1c	0.91de	1223.7***
**Water Potential**							
Shoot water potential (-Mpa)	1.4c	1.5b	1.6a	1.6a	1.4c	1.5b	169.1***
Shoot osmotic potential (-Mpa)	1.2a	1.2a	1.1b	1.2a	0.8c	0.6d	21.7***
Shoot turgor potential (Mpa)	0.24d	0.29 cd	0.48bc	0.38c	0.56b	0.92a	18.7***
**Ionic Contents**							
Shoot Na^+^ (mg g^−1^ d.wt.)	21.2f	25.5d	24e	32.5a	30.2b	27.7c	11.4***
Root Na^+^ (mg g^−1^ d.wt.)	16.5b	14c	14.5c	15.2bc	18.5a	18.2a	3.8*
Shoot Ca^2+^ (mg g^−1^ d.wt.)	20.5b	21.5ab	15.3d	19 cd	18.3c	22.5a	3.3*
Root Ca^2+^ (mg g^−1^ d.wt.)	4.2b	4.1b	2.9d	3.7c	4.5a	4.3a	2.2^NS^
Shoot K^+^ (mg g^−1^ d.wt.)	8d	10b	9.8c	8.5 cd	15a	10.5ab	13.4***
Root K^+^ (mg g^−1^ d.wt.)	22d	27.5c	30.5b	27.5c	34.2a	29.5bc	10.1***
**Organic Osmolytes**	645.7d	1283.3c	1900ab	1895.8ab	1950a	1726.7b	35.5***
Total free amino acids (µg g^-1^f. wt.)							
Proline (µmol g^-1^f. wt.)	102de	113.9d	190.4b	187b	255a	136c	9.3**
Total soluble proteins (µg g^-1^f. wt.)	948bc	600d	732 cd	1020a	960b	792c	1.4^NS^
Total soluble sugars (mg g^−1^ d. wt.)	27.7b	26.3bc	25.3c	30.1a	27b	27.4b	0.97^NS^

Means sharing similar letters in each row are statistically not significant.

* = Significant at p < 0.05, ** = significant at p < 0.01, *** = significant at p < 0.001, NS = not significant.

### 3.2 Water relation traits

Jhalar Lake and Pakka Anna exhibited highest values of shoot water potential, which have no significant variation from other collection sites of *C. laevigatus* (Table 2). The maximum shoot osmotic potential was reported in Haroonabad, Khushab and Pakka Anna populations, with minimum values observed in Sahianwala population. Sahianwala surpassed all the other populations regarding shoot turgor potential. All other habitats varied significantly, with least potential observed in Haroonabad.

### 3.3 Ionic content

Pakka Anna population was reported with highest Na^+^ contents in shoots. It showed variation from 21.2 to 32.5 mg g^−1^ d.wt., with least values noted in Haroonabad (Table 2). Root Na^+^ ranges in quantity from 18.5 to 14 mg g^−1^ d.wt. in Kalar Kahar Lake and Khushab respectively. This character is not much diverse among different habitats of *C. laevigatus.* Shoot and root Ca^2+^ was found the maximum in population of Sahianwala, with the minimum values observed in Jhalar Lake. There was no significant difference among habitats regarding root Ca^2+^. Kalar Kahar Lake exhibited the highest shoot K^+^ and root K^+^ with slight variation among other populations of *C. laevigatus.* Population of Haroonabad was recorded with the least values of shoot K^+^ and root K^+^.

### 3.4 Organic osmolytes

Total free amino acids and proline accumulate maximally in population collected from Kalar Kahar Lake (Table 2). Free amino acids differ notably among other habitats, with least values noted in Haroonabad. The saline habitat, Pakka Anna surpassed all the other habitats of *C. laevigatus* regarding soluble proteins and sugars but showed no significant variation among selected sites.

### 3.5 Root anatomical characteristics

**Fig. 3:**
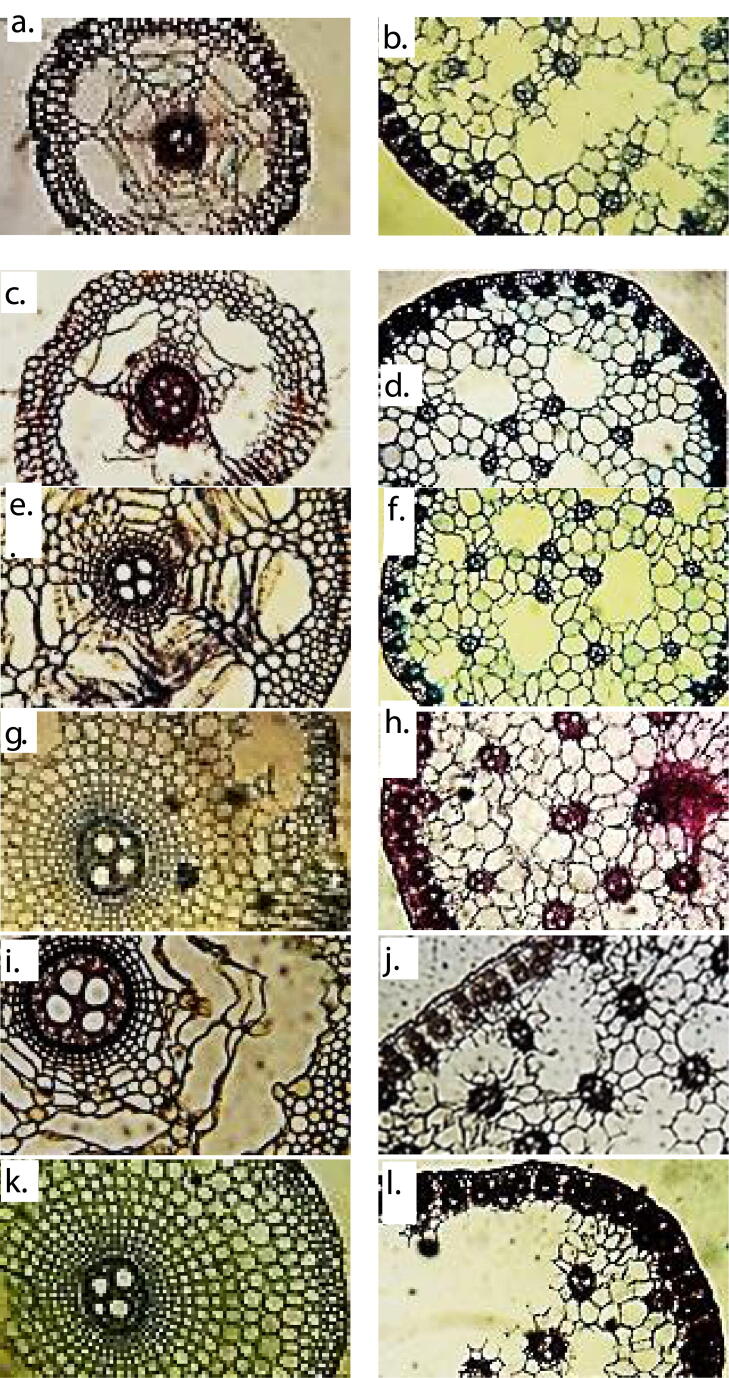
Root and stem anatomical characteristics of *Cyperus laevigatus* collected from different ecological zones of Punjab. **a. Haroonabad Bract TS:** Vascular bundles towards periphery, chlorenchyma with sclerification, large cortical cells, larger aerenchyma. **b. Haroonabad Leaf sheath TS:** Moderately thick leaf sheath, smaller vascular bundles, reduced aerenchyma, sclerification under upper epidermis. **c. Khushab Bract TS:** Reduced bract with smaller aerenchyma and vascular bundles, smaller cortical cells. **d. Khushab Leaf sheath TS:** Comparatively thick leaf sheath, larger aerenchyma, minor sclerification under upper epidermis, lower epidermis one-cell layered. **e. Jhalar lake Bract TS: S**mall epidermal cells, vascular bundles towards half periphery. **f. Jhalar lake Leaf sheath TS:** Very thick leaf sheath, larger vascular bundles, very large aerenchyma, sclerification under upper epidermis and two-cell layered lower epidermis. **g. Pakka anna Bract TS:** Peripheral and central vascular bundles, many aerenchyma, small and large cortical cells. **h. Pakka anna Leaf sheath TS:** squished leaf sheath at some points, larger aerenchyma, vascular bundles of varied size. **i. Kalar Kahar lake Bract TS:** Both central and peripheral vascular bundles, one large & two small aerenchyma. **j. Kalar Kahar lake Leaf sheath TS:** Thick leaf sheath, small and large vascular bundles, little sclerification under upper epidermis. **k. Sahianwala Bract TS:** vascular bundles towards periphery, larger cortical cells, no aerenchyma. **l. Sahianwala Leaf sheath TS:** Comparatively thin leaf sheath, larger vascular bundles, small aerenchyma, some larger cortical cells.

Root anatomical features varies significantly among selected habitats of *C. laevigatus* (Table 3 & Fig. 3). Root and cortical thickness was observed the maximum in Kalar Kahar Lake population, while least thickness recorded in Haroonabad. Population collected from Haroonabad depicted thickest epidermis of root along with thinnest vascular region and the least aerenchyma area. The maximum endodermal thickness, metaxylem area, vascular region thickness and aerenchymatous area reported in populations of Kalar Kahar Lake. Khushab population depicted the minimum cortical cell size, endodermal thickness and vessels size. Population collected from Pakka Anna surpassed all other collection sites regarding cortical cell area. The Jhalar Lake was observed with minimum values of Epidermal thickness.

**Table 3:** Anatomical characteristics of *Cyperus laevigatus* collected from different ecological regions of Punjab.

	Har	Khu	Jha	PA	KK	Sah	F-ratio
**Root Anatomy**							
Root thickness (µm)	261.4 cd	310.5c	449.3bc	473.9b	620.9a	433.0bc	81.2***
Epidermal thickness (µm)	16.3a	15.5ab	6.5 cd	8.9c	15.5ab	12.2b	72.8***
Cortical thickness (µm)	98.0 cd	106.2c	196.1ab	204.2ab	1253.3a	179.7b	162.5***
Cortical cell area (µm^2^)	288.8e	209.6f	504.9c	629.7a	440.9d	587.7b	85.9***
Endodermal thickness (µm)	7.3d	4.1f	8.9b	6.5e	9.8a	8.2c	18.5***
Metaxylem area (µm^2^)	94.7d	47.5de	210.1c	334.9b	551.0a	199.6c	196.3***
Vascular region thickness (µm)	15.5d	23.7c	17.9 cd	34.3b	44.9a	31.9bc	554.0***
Aerenchymatous area (µm^2^)	2311.1c	2646.7bc	3150.2b	0	4618.7a	0	140.6***
**Stem Anatomy**							
Stem thickness (µm)	547.4 cd	637.3bc	580.1c	612.7bc	645.4b	743.5a	22.7***
Epidermal thickness (µm)	12.2b	10.6bc	8.2 cd	17.1a	15.5ab	8.9c	58.1***
Cortical cell area (µm^2^)	2014.3b	1410.1de	1783.5c	1636.6 cd	2360.4a	1516.5d	96.3***
Vascular bundle area (µm^2^)	1038.8d	752.9e	734.6ef	2403.4b	1737.3c	2634.2a	630.3***
Metaxylem area (µm^2^)	58.0b	59.1b	75.9a	44.4c	68.5ab	52.3bc	2.9^NS^
Sclerenchyma thickness (µm)	8.9f	20.4b	23.7a	17.9c	15.5d	11.4e	136.80***
Chlorenchyma thickness (µm)	25.3bc	23.7c	20.4 cd	38.4ab	42.5a	32.7b	346.40***
Stomatal area (µm^2^)	419.9e	629.7c	797.5b	588.8d	918.1a	920.8a	75.85***
Stomatal density	14.7c	11.7 cd	19.7b	16.7bc	18bc	27a	34.35***
**Bract Anatomy**	650.9a	160.7d	294.1bc	291.4bc	253.3c	422.1b	187.7***
Bract thickness (µm)							
Epidermal thickness (µm)	11.2c	8.4d	16.6ab	17.9a	16.3ab	15.2b	23.9***
Cortical cell area (µm^2^)	1007.3b	639.1 cd	818.5c	734.6bc	755.6bc	2242.4a	473.1***
Vascular bundle area (µm)	671.6e	1442.6ab	1463.6a	760.8d	1091.2c	1321.9b	147.2***
Metaxylem area (µm^2^)	26.0 cd	50.7ab	52.3a	29.7c	10.8d	42.3b	10.4***
Chlorenchyma thickness (µm)	21.3c	22.7c	18.4d	38.5a	28.4b	29.7b	11.4***
Aerenchyma area (µm^2^)	3410.4ab	3092.5b	4041.8a	2835.6c	1765.7d	0	57.7***
**Leaf Sheath Anatomy**	85.8 cd	73.5d	163.4a	89.9c	130.7b	81.7 cd	55.72***
Leaf sheath thickness (µm)							
Upper epidermal thickness (µm)	8.9e	15.5b	12.2d	14.7c	16.3a	8.2ef	53.70***
Lower epidermal thickness (µm)	12.2c	14.7b	8.9de	9.8d	16.3a	7.3e	54.50***
Cortical Cell Area (µm^2^)	503.8a	231.1 cd	219.6d	262.6c	283.5bc	385.8b	53.71***
Aerenchyma Area (µm^2^)	3543.6 cd	3674.7c	10073.0a	4199.1bc	5772.5b	2206.2d	112.78***
Vascular bundle area (µm^2^)	472.4d	903.5c	1564.3a	1070.2bc	866.7 cd	1101.7b	173.77***
Metaxylem area (µm^2^)	10.8f	16.1e	42.3b	52.8a	26.0d	33.4c	11.94***

Means sharing similar letters in each row are statistically not significant.

* = Significant at p < 0.05, ** = significant at p < 0.01, *** = significant at p < 0.001, NS = not significant.

### 3.6 Stem anatomical characteristics

The thickest stem was reported in population of Sahianwala and epidermis in population of Pakka Anna (Table 3 & Fig. 3). Population of Haroonabad showed the minimum stem thickness and stomatal area. Cortical cell area, chlorenchyma thickness and stomatal area was reported the maximum in Kalar Kahar Lake population (Table 3 & Fig. 5). Khushab population depicted lowest cortical cell area and least number of stomata. Vascular bundle area, stomatal area and stomatal density values were recorded the maximum in Sahianwala population, with least thickness of sclerenchymatous tissues. The narrowest vessel was observed in population of Pakka Anna among all the selected habitats. The minimum values for epidermal thickness, vascular bundle area and chlorenchyma thickness was observed in Jahla Lake population. Broader vessels and thicker sclerenchyma tissues were also noted in population of this habitat.

### 3.7 Bract anatomical characteristics

**Fig. 4:**
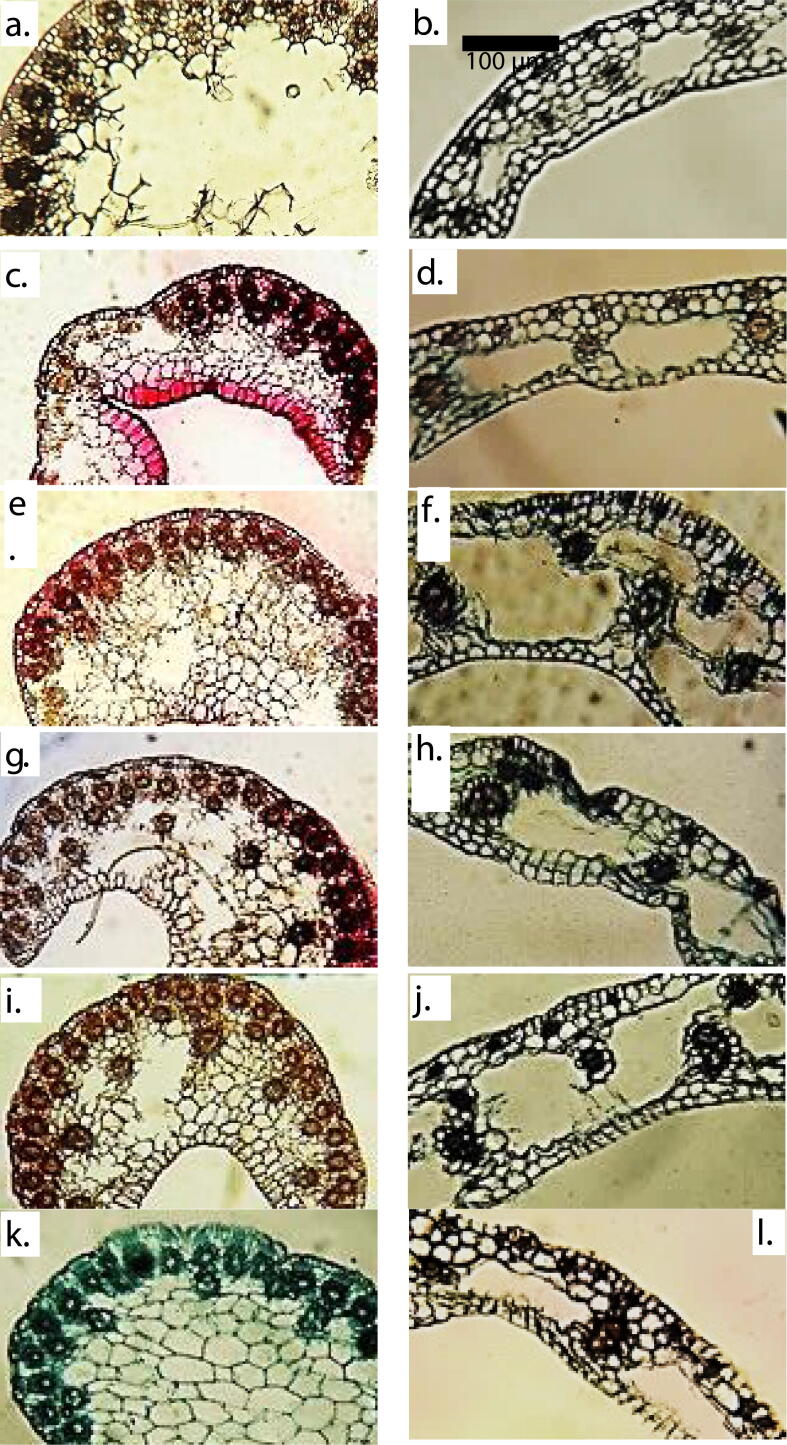
Bract and Leaf sheath anatomical characteristics of *Cyperus laevigatus* collected from different ecological zones of Punjab. **a. Haroonabad Stem epidermis:** Dum-bell shaped larger stomata with parallel rows of larger epidermal cells. **b. Khushab Stem epidermis:** Stomata arranged in rows, comparatively small. **c. Jhalar lake Stem epidermis:** Stomata and epidermal cells stained alike, some epidermal cell smaller and others large. **d. Pakka anna Stem epidermis:** Darkly stained stomata arranged in rows. **e. Kalar Kahar lake Stem epidermis:** Smaller stomata with larger epidermal rows. **f. Sahianwala Stem epidermis:** Larger stomata, smaller and larger epidermal rows.

Haroonabad population possessed the highest values for bract thickness and lowest values for vascular bundle size and chlorenchyma thickness (Table 3 & Fig. 4). Population collected from Sahianwala surpassed all other habitats regarding cortical cell area. Narrow vessels and smaller aerenchyma observed in Kalar Kahar Lake population. Epidernal and chlorenchyma thickness was reported the maximum in Pakka Anna population. Population of Jhalar Lake depicted larger vascular bundles, broader vessels and the maximum sized aerenchyma. Bract thickness, epidermal thickness and cortical cell area recorded with the minimum values in population of Khushab.

**Fig. 5:**
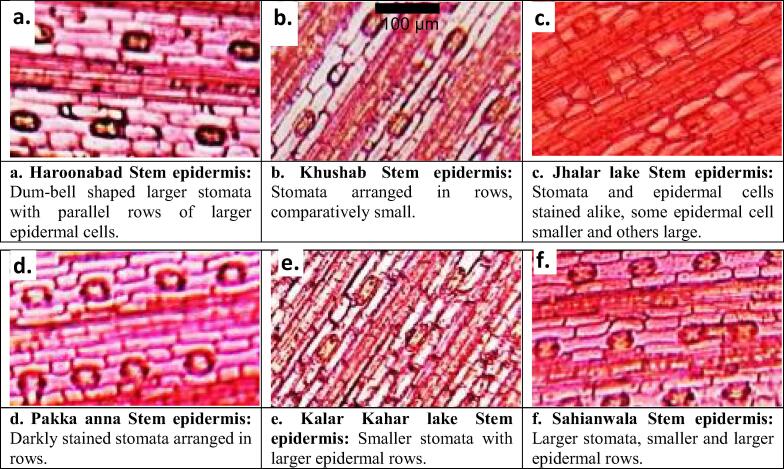
Stem epidermal characteristics of *Cyperus laevigatus* collected from different ecological zones of Punjab.

### 3.8 Leaf sheath anatomical characteristics

Population of Jhalar lake had leaf sheath with the maximum thickness as compared to other collection sites (Table 3 & Fig. 4). The maximum values for aerenchyma area and vascular bundle area were also noted in this site, along with smallest cortical cells. Broader and narrower vessels reported in populations of Pakka Anna and Haroonabad respectively. Upper and lower epidermal thickness was recorded with higher values in population of Kalar Kahar Lake. Sahianwala population possessed the minimum aerenchyma area, upper and lower leaf sheath thickness. The thinnest leaf sheath was observed in population collected from riverbank of Khushab. Haroonabad population showed maximum cortical cell area and minimum vascular bundle area.

### 3.9 Relationship among habitats and plant structural & functional attributes

**Fig. 6:**
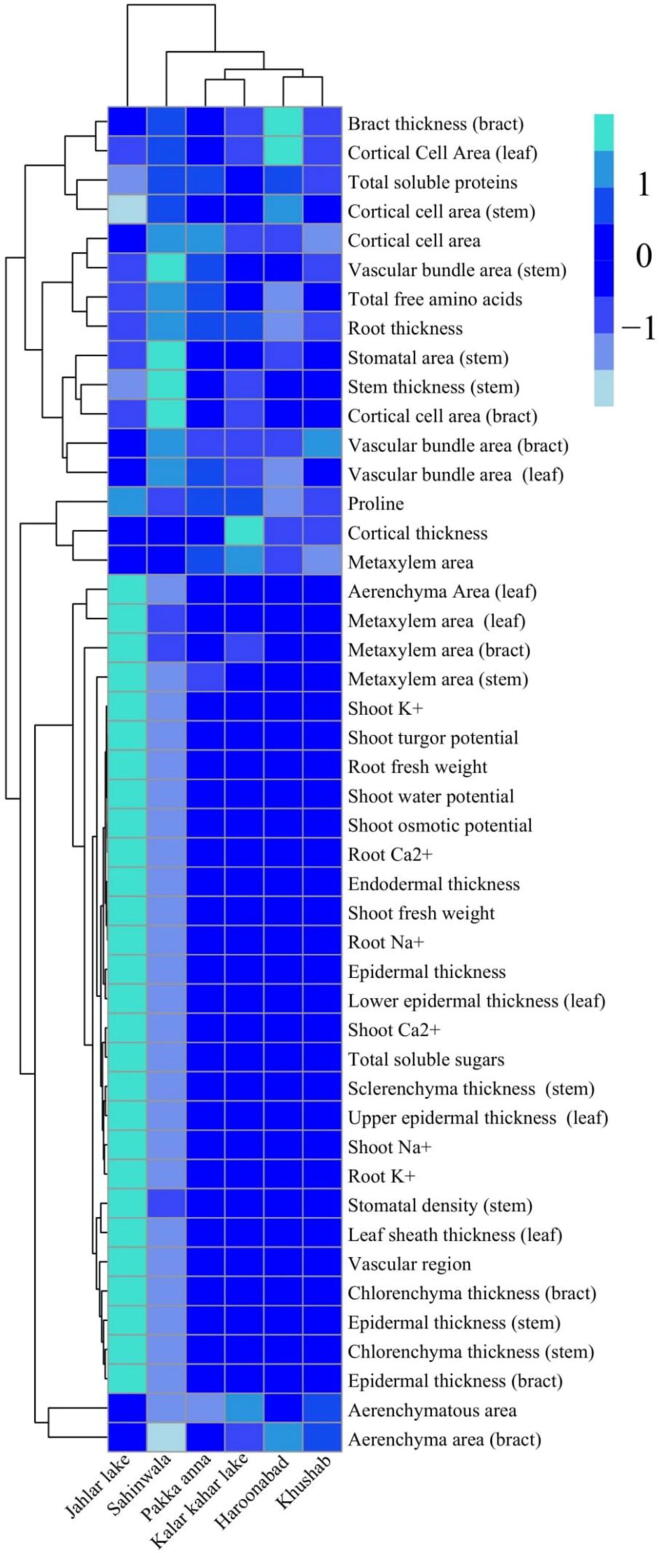
Heat-map analysis between different growth, osmolytes, ions uptake and anatomical changes in *Cyperus laevigatus* collected from different ecological zones of Punjab.

A heat-map analysis was constructed to quantify the relationship between different morpho-physiological and anatomical traits of *C. laevigatus* collected from different regions of Punjab district are presented in Fig. 6. Almost most of parameters are showing no relationship or negative relationship with their habitat. However, some growth parameters, ions, osmolytes and anatomical traits showed a significant positive relationship which were collected from Jahlar lake. In this heat-map analysis blue colour is indicating no significant difference while turquoise is showing a significant postive relationship with selected habitats. This relationship is showing a close connection between different parameters of *Cyperus laevigatus* to selected habitats of Punjab.

### 3.10 Association between soil and plant morpho-physiological & anatomical parameters

**Fig. 7:**
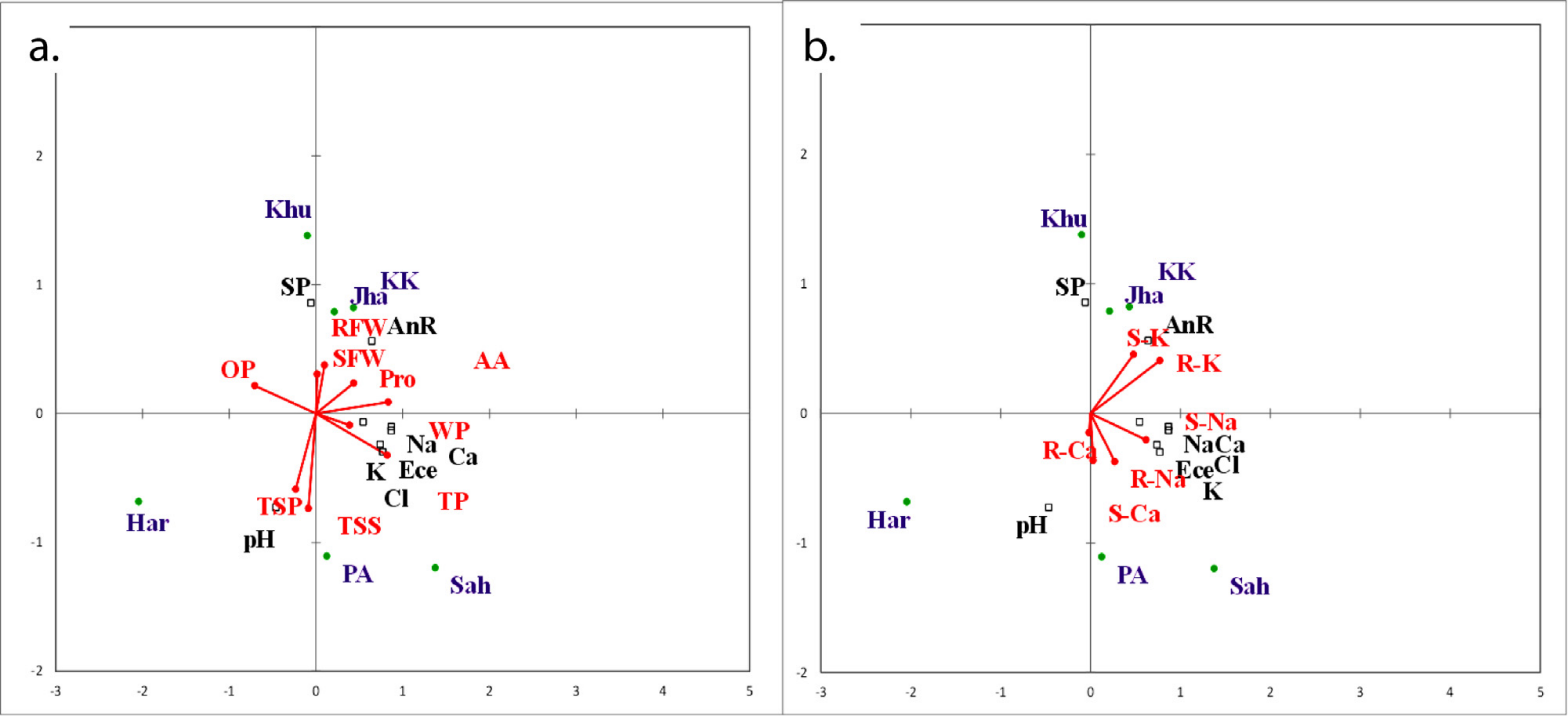
RDA Ordination biplot showing effect of soil physico-chemical characteristics on (a) morphological and (a & b) physiological characteristics of *Cyperus laevigatus* collected from different habitats (Har: Haroonabad; Khu: Khushab; Jha: Jahlar Lake, PA: Pakka anna, KK: Kalar Kahar Lake, Sah: Sahianwala; AnR: Annual rainfall; SP: Saturation percentage; SFW: Shoot fresh weight; RFW: Root fresh weight; WP: Shoot water potential; OP: Shoot osmotic potential; TP: Shoot turgor potential; S-Na: Shoot Na+; R-Na: Root Na+; S-Ca: Shoot Ca2+; R-Ca: Root Ca2+; S-K: Shoot K+; R-K: Root K+; AA: Total free amino acids; Pro: Proline; TSP: Total soluble proteins; TSS: Total soluble sugars).

**Fig. 8:**
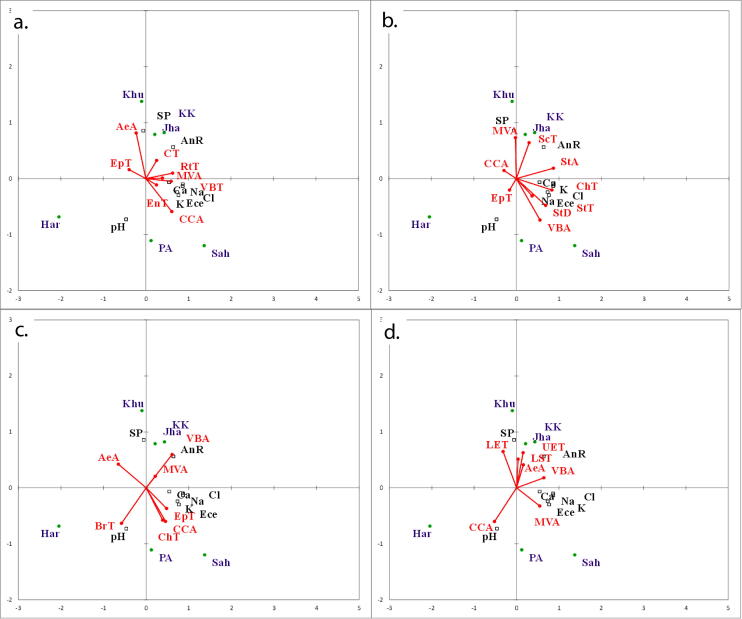
RDA Ordination biplot showing effect of soil physico-chemical characteristics on (a) root (b) stem (c) bract and (d) leaf sheath anatomical characteristics of *Cyperus laevigatus* collected from different habitats (Har: Haroonabad; Khu: Khushab; Jha: Jahlar Lake, PA: Pakka anna, KK: Kalar Kahar Lake, Sah: Sahianwala; AnR: Annual rainfall; SP: Saturation percentage; RtT: Root thickness; EpT: Epidermal thickness; CT: Cortical thickness; CCA: Cortical cell area; EnT: Endodermal thickness; MVA: Metaxylem area; VBT: Vascular region thickness; AeA: Aerenchymatous area; StT: Stem thickness; VBA: Vascular bundle area; ScT: Sclerenchyma thickness; ChT: Chlorenchyma thickness; StA: Stomatal area; StD: Stomatal density; BrT: Bract thickness; LST: Leaf sheath thickness; UET: Upper epidermal thickness; LET: Lower epidermal thickness).

Redundancy analysis (RDA) ordination biplot illustrated the impact of soil characteristics of diverse habitats on physiological and morpho-anatomical attributes of *Cyperus laevigatus* (Fig. 7 & Fig. 8). Root fresh weight and sheet fresh weight strongly associated with annual rainfall at Jahlar and Kalar Kahar lake. Water and turgor potential possessed association with soil EC, Na^+^, Ca^2+^, K^+^ and Cl^-^ contents. Shoot and root K^+^ associated strongly with annual rainfall. Root Ca^2+^ depicted relationship with soil pH. Root thickness, cortical thickness and metaxylem area possessed strong association with annual rainfall at Kalar Kahar and Jahlar lake. Root aerenchyma area had association with Khushab. Stem thickness, vascular bundle area, chlorenchyma thickness and stomatal density exhibited strong relationship with soil EC, Na^+^, Ca^2+^, K^+^ and Cl^-^ contents at Sahianwala. Bract thickness associated with soil pH at Haroonabad. Epidermal thickness, cortical cell area and chlorenchyma thickness associated strongly with soil EC, Na^+^, Ca^2+^, K^+^ and Cl^-^ contents at Pakka Anna. Leaf sheath thickness, aerenchyma area and vascular bundle area had strong relationship with rainfall at Jahlar and Kalar Kahar lake. Lower epidermal thickness showed association with saturation percentage at Khushab.

## 4 Discussion

Ecotypic variation can be determined by investigating structural and functional modifications of different populations of a species (Rana et al., 2020a). Structural features are most vulnerable to surrounding climatic factors and strongly respond to abiotic stresses (Ali et al., 2020b, Rehman et al., 2020, Saleem et al., 2020a). Each population of *Cyperus laevigatus* showed different structural and functional response, it can be determined that adaptive characteristics have been fixed during evolutionary process and this can be a reason behind variable developmental behavior (Badr et al., 2020).

Haroonabad is a non-saline habitat, located at edge of Cholistan desert with smaller annual rainfall and soil with good saturation percentage. Higher osmotic potential in this population leads to better growth and biomass production (Hussain et al., 2016). K^+^ ions and osmolytes found in very low quantity because these plants are not facing any environmental stress. Water conservation is not the main strategy of Haroonabad population because its not facing the physiological drought caused by salinity (Corrêa et al., 2017). This population exhibited less development of vascular tissues, aerenchyma and root cortex which indicates non-stressful environment of this habitat (Mohamed et al., 2020a). Stomatal area of this population is much decreased which may be beneficial during less availability of water (Khan et al., 2019). Larger cortical cells of leaf sheath maintain turgor of plants when they face water scarcity (Liang et al., 2018, Nazar et al., 2020).

Saline soil of Khushab reduced biomass production of *C. laevigatus*. It receives good annual rainfall but due to dry barren hills and salinity enough water is not available to plants. A moderate accumulation of K^+^ ions in this population may be for K^+^/Na^+^ selectivity for preventing excessive Na^+^ ion uptake (Alam et al., 2020). No noticeable alteration observed in anatomical features of this population but narrow xylem vessels are highly advantageous because wide vessels can be susceptible to collapse under physiological drought (Goharrizi et al., 2020, Rana et al., 2020b). Low stomatal density exhibited adaptation of this population to dry barren land, which is very effective for water conservation (Khan et al., 2019, Afzal et al., 2020).

Jahlar lake is a salt-water lake situated in mountains, dominated with sedges and salt-tolerant grasses. It receives moderately high annual rainfall. Maximum shoot water potential of this population contributes to the highest shoot and root fresh weight (Hussain et al., 2018). The thinnest root epidermis observed because water conservation not required due to availability of plenty of water. Broader vessels in vascular bundles involved in effective water movement when availability of water is sufficient (Ahmad et al., 2017). Aerenchyma formation in bracts and leaf sheath provide benefits to this population for supply of oxygen under hypoxic conditions. Aerenchyma development has been recorded in populations growing under salt stress (Mohamed et al., 2020b). Periphery of stem is highly sclerified that has contribution to water conservation as well as mechanical strength to plants (Gunes et al., 2006).

Pakka Anna is a hyper-saline wetland that is dominated by halophytes, receives sufficient annual rainfall. Plants were poorly developed with minimum shoot fresh weight most probably due to physiological stress caused by saline wetland and highest accumulation of Na^+^ ions in shoots (Parihar et al., 2015). Under salt stress, soluble proteins and sugars accumulated excessively to reduce the adverse effects of ROS (Ali et al., 2020a). Thick epidermal cells of stem minimize the water loss under shortage of water during physiological stress. Large cortical cells of root mostly possess larger vacuoles that may be advantageous for physiological drought under waterlogged soils (Ahmad et al., 2017). Enhanced chlorenchyma thickness observed in bract to fulfil the photosynthetic requirement because salt stress hinder the physiological mechanisms (Gunes et al., 2006).

Kalar Kahar is a hyper-saline lake, receives maximum annual rainfall as compared to other habitats. Population collected from the bank of lake exhibited ultimate tolerance to salt stress. There is excessive accumulation of Na^+^ contents in roots, toxicity of these ions nullified by the accumulation of Ca^2+^ and K^+^ ions (Zamin and Khattak, 2017). Higher accumulation of osmolytes like free amino acids and proline reported, that reveals high level tolerance to salt stress in population of Kalar Kahar (Szabados and Savouré, 2010). This population exhibited the maximum of mostly anatomical features like dermal thickness, mechanical tissues, vascular and storage tissues. All these anatomical features are essential for water conservation either by storage of water or by preventing water loss from plant body (Abid et al., 2018).

Sahianwala is located near Faisalabad, highly saline waterlogged salt marsh. It receives moderate annual rainfall, but saline soil restricted the biomass production of *C. laevigatus.* Higher accumulation of Ca^2+^ contents may take part in the neutralization of side effects caused by salt stress (Kamran et al., 2019). Stem growth observed maximum in this population to store the maximum amount of water in parenchymatous tissues for stressed environmental conditions (Hasanuzzaman et al., 2018). Larger vascular bundles with broader xylem vessels associated with enhanced water conductivity (Mohamed et al., 2020b). Increased stomatal size in this population may associated with efficient photosynthetic process (Kamran et al., 2019).

## 5 Conclusion

*Cyperus laevigatus* ecotypes exhibited higher level of plasticity in structural and functional features, which offer this species a great ability to flourish in variable stressed habitats. Populations of this species were collected from different saline habitats like dry barren soils, saline lakes, hyper-saline wetlands and salt marshes to evaluate anatomical modifications and ionic homeostasis. Each population revealed specific adaptations regarding anatomical and physiological characteristics, which exhibited its adaptability potential to harsh environmental conditions. Population of Jahlar lake showed maximum biomass production indicates that it grows better in moderate salinities. This species will prove very useful for revegetation of salt affected rangeland and prairies by direct growth of such halophytic ecotypes. Genes can be extracted from this species and incorporated to crops to enable them to grow in high salinities either by conventional breeding or advanced molecular biology approaches.

**Ethics approval**

Not Applicable

**Consent to participate**

All authors consent to participate in this manuscript.

**Consent for publication**

All authors consent to publish this manuscript in Saudi journal of Biological Science

**Availability of data and material**

Data will be available on request to the corresponding or first author

**Code availability**

Not Applicable

**Authors' contributions**

Sahar Mumtaz, Mansoor Hameed, Athar Mahmood conceived and designed the study and Fatima Batool, Syeda Fasiha Amjad and Abida Parveen critically revised the manuscript and approved the final version. Shakeel Ahmed, Abdulaziz Abdullah Alsahli, Mohammed Nasser Alyemeni and Humaira Yasmin executed the experiment and compiled data. Muhammad Hamzah Saleem supervised the experiment and Muhammad Arfan helped in sample collection and chemical analysis. All authors read and approve the same for publication.

## Declaration of Competing Interest

The authors declared that there is no conflict of interest.
